# Comparative Transcriptome Analysis of Climacteric Fruit of Chinese Pear (*Pyrus ussuriensis*) Reveals New Insights into Fruit Ripening

**DOI:** 10.1371/journal.pone.0107562

**Published:** 2014-09-12

**Authors:** Guohui Huang, Tong Li, Xinyue Li, Dongmei Tan, Zhongyu Jiang, Yun Wei, Juncai Li, Aide Wang

**Affiliations:** 1 College of Horticulture, Shenyang Agricultural University, Shenyang, China; 2 Department of Horticulture, Eastern Liaoning University, Dandong, China; 3 Division of Pear Breeding, Institute of Pomology, Liaoning Academy of Agricultural Sciences, Xiongyue, China; Key Laboratory of Horticultural Plant Biology (MOE), China

## Abstract

The fruit of *Pyrus ussuriensis* is typically climacteric. During ripening, the fruits produce a large amount of ethylene, and their firmness drops rapidly. Although the molecular basis of climacteric fruit ripening has been studied in depth, some aspects remain unclear. Here, we compared the transcriptomes of pre- and post-climacteric fruits of Chinese pear (*P. ussuriensis* c.v. Nanguo) using RNA-seq. In total, 3,279 unigenes were differentially expressed between the pre- and post-climacteric fruits. Differentially expressed genes (DEGs) were subjected to Gene Ontology analysis, and 31 categories were significantly enriched in the groups ‘biological process’, ‘molecular function’ and ‘cellular component’. The DEGs included genes related to plant hormones, such as ethylene, ABA, auxin, GA and brassinosteroid, and transcription factors, such as *MADS*, *NAC*, *WRKY* and *HSF*. Moreover, genes encoding enzymes related to DNA methylation, cytoskeletal proteins and heat shock proteins (HSPs) showed differential expression between the pre- and post-climacteric fruits. Select DEGs were subjected to further analysis using quantitative RT-PCR (qRT-PCR), and the results were consistent with those of RNA-seq. Our data suggest that in addition to ethylene, other hormones play important roles in regulating fruit ripening and may interact with ethylene signaling during this process. DNA methylation-related methyltransferase and cytoskeletal protein genes are also involved in fruit ripening. Our results provide useful information for future research on pear fruit ripening.

## Introduction

There are three ecotypes of Chinese pear: *Pyrus bretschneideri*, *Pyrus pyrifolia* and *Pyrus ussuriensis*. *P. ussuriensis* grows in the cold, northern part of China, and its fruits undergo typical respiratory climacteric during ripening. Climacteric is the final physiological step that marks the onset of fruit ripening, and it results in the increase of internal and external characteristics, such as flavor, nutritional value, sugars, acids and pigment. The defining point of climacteric is the burst of ethylene production in the fruit, and this process usually occurs without any external influence [1].

The plant hormone ethylene plays critical roles in the ripening process of climacteric fruit. Extensive knowledge of ethylene signal transduction and response has been gained from studies on tomato [1]. In addition to ethylene, other hormones play important roles in fruit ripening. In tomato, the level of auxin (indole-3-acetic acid, IAA) is decreased before fruit ripening [2]. Two members of the *GH3* (*IAA-amino synthase*) gene family have been associated with the ripening of grapes [3]. Little is known about the role of GA (gibberellin) in fruit ripening, but it has been reported that the external application of GA3 to ripening strawberry fruits delayed the development of red color [4]. Abscisic acid (ABA) is known to be involved in the ripening of climacteric fruit. In tomato, suppressing the *NCED1* (*9-cis-epoxycarotenoid dioxygenase*) gene, which encodes the key enzyme in ABA biosynthesis, decreases the expression of ripening-related genes such as PG (polygalacturonase) and extends the fruit's shelf life [5]. Many studies have been conducted on BR (brassinosteroid), but its role in fruit ripening remains unclear.

The transcriptional regulation of fruit ripening has primarily studied through studies on the tomato *RIN* (*Ripening inhibitor*) gene [1]. *RIN* is a *MADS-box* transcription factor gene, and its deletion inhibits the ripening of tomato fruits [6]. Genes involved in ethylene biosynthesis, such as *1-aminocyclopropane-1-carboxylic acid synthase* (ACS) and *1-aminocyclopropane-1-carboxylic acid oxidase* (ACO), are under the regulation of RIN [7]. In apple, the expression levels of *MdACS1* and *MdACO1* are greatly decreased in transgenic lines in which the *MADS-box* gene *MdMADS8* is silenced, indicating that *MADS-box* genes play critical roles in ethylene biosynthesis [8]; moreover, *MdMADS2* gene expression has been shown to be associated with fruit firmness [9]. A NAC domain transcription factor has also been reported to be involved in fruit ripening, possibly operating upstream of the *MADS-box* genes [10]. Although much knowledge has been gained from studying *MADS-box* and *NAC*, it remains unclear whether other transcription factors are involved in fruit ripening.

It has also been reported that DNA methylation is related to fruit ripening [1]. A well-known example is the tomato *Colorless non-ripening* (*Cnr*) mutant. The fruit of *Cnr* show green color and failure to ripen, and a high level of cytosine methylation is observed in the promoter region of the *Cnr* gene, leading to the non-ripening phenotype [11]. A more recent study found that the promoter region of *Cnr* was demethylated just before the initiation of fruit ripening, indicating the influence of DNA methylation on fruit ripening [12]. Current reports have focused solely on DNA methylation changes during development; the role of DNA methylation-related enzymes in fruit ripening has not yet been documented.

Although considerable knowledge has been gained from studies of ethylene and other hormones in fruit ripening, it is believed that other, unknown factors may influence fruit ripening [13]. Moreover, the ripening behavior of *P. ussuriensis* has not previously been studied. A total transcriptome analysis of pre- and post-climacteric fruits of *P. ussuriensis* would provide insights into the ripening process of pear fruits and identify new candidates for genes regulating climacteric pear fruit ripening. In this study, we analyzed the transcriptomes of *P. ussuriensis* fruits during pre- and post-climacteric stages. Several plant hormones, including ethylene, and a few transcription factors, in addition to *MADS-box* genes, were differentially expressed between pre- and post-climacteric fruits. Other factors, such as DNA methylation-related enzymes and cytoskeletal protein genes, showed differential expression during fruit ripening. In addition, the expression of select differentially expressed genes was confirmed by quantitative RT-PCR (qRT-PCR).

## Methods

### Plant materials

Fruits of ‘Nanguo’ pear (*P. ussuriensis*) were harvested on Sep. 13, 2012, from the experimental farm of Liaoning Institute of Pomology (Xiongyue, China). All of the fruits were stored at room temperature (RT, 24°C) for 15 d and sampled every 5 d. At each sampling point, 5 fruits were collected and subjected to firmness and ethylene-production measurements as described by Li et al. [14]. The extraction of fruit juice and the measurement of titratable acid were performed as described by Xu et al. [15]. The total soluble solids were measured from the above fruit juice of each sample using a Brix tester (PAL-1, ATAGO, Japan). The flesh of 5 fruits were sliced, pooled and frozen in liquid N_2_ and stored at −80°C for RNA extraction.

The treatment of fruits with 1-MCP (an ethylene antagonist) was performed according to Li et al. [14]. ‘Nanguo’ pear fruits collected at commercial harvest time (0, Pre) and at 10 d after storage (10, Post) were used for RNA-seq. Each sample was sequenced twice, and a total of 4 samples were used for RNA-seq.

### RNA extraction and deep sequencing

Total RNA extraction was performed as described by Li et al. [14]. mRNA was isolated from total RNA using beads with Oligo(dT), and 10× fragmentation buffer was used to cut the mRNA into short fragments. First-strand cDNAs were synthesized with random primers using the short fragments of mRNA as templates. Second-strand cDNAs were synthesized with DNA polymerase I (TaKaRa) and purified with a QIAquick PCR Purification Kit (Qiagen). The purified cDNAs were subjected to end reparation and polyadenylation and were mixed with Solexa adapters. Suitable fragments were recovered from an agarose gel and amplified by PCR, and they were then sequenced using an Illumina HiSeq 2000.

### Bioinformatics analysis

Low-complexity reads and the reads containing adapter sequences were removed from the raw reads, and the resulting clean reads were mapped to the reference genome using TopHat software [16]. Unigenes were first aligned by BLASTx (*E* value <10^−7^) to the NCBI non-redundant protein database [17]. The analysis of differentially expressed genes (DEGs) was performed using the method described by Trapnell et al. [18], and the false discovery rate (FDR) was used to determine the P-value thresholds via multiple testing [19]. The FPKM (reads per kb per million reads) method was used to calculate the rate of DEGs [20], with a *p* value ≤0.05 and a fold change value ≥2. Gene Ontology (GO) analysis was applied to the DEGs, and a Bonferroni correction-corrected *P* value ≤0.05 was defined as significant enrichment. The heat map for DEGs involved in ethylene biosynthesis and signaling pathway was constructed using Cluster 3.0. All the raw data has been deposited into NCBI Sequence Read Archive (SRA) under accession number SRP045291.

### qRT–PCR

First-strand cDNA was synthesized from 500 ng of total RNA using the M-MLV RTase cDNA Synthesis Kit (Cat#D6130, TaKaRa) and was diluted 10 times with H_2_O and then used as templates for qRT-PCR assays. qRT-PCR was conducted as described by Tan et al. [21]. Specific primers for each gene were designed using Primer3 (http://frodo.wi.mit.edu/) and are listed in Table S8. The pear *beta*-tubulin [22], actin1 (accession number AB190176) and actin2 (AF386514) genes, which showed no differential expression in this study, were used as internal controls. The geometric mean of these three reference genes was used to normalize the expression of the target genes. Three replications were conducted.

## Results

### Sample preparation and RNA-seq of ‘Nanguo’ pear pre- and post-climacteric fruits

**Figure 1:**
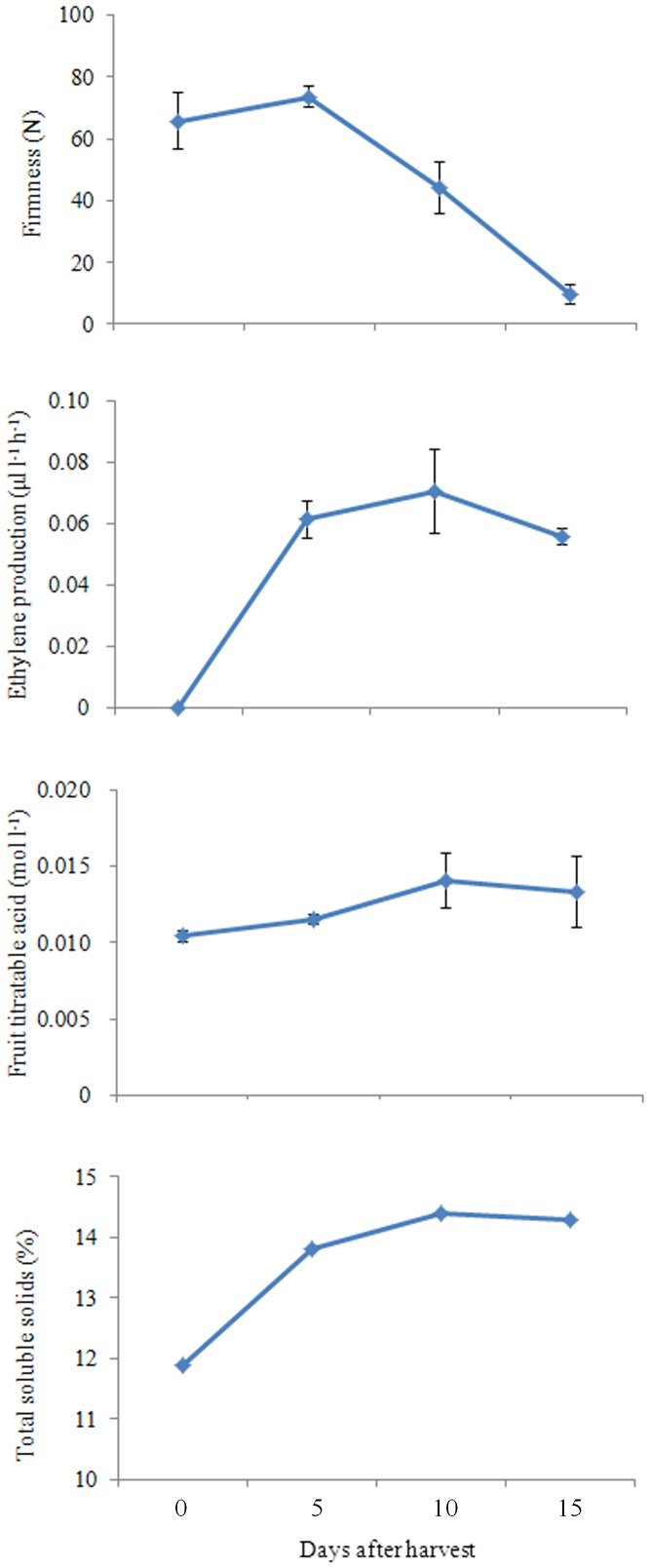
Fruit firmness, ethylene production, titratable acid and total soluble solids of ‘Nanguo’ pear fruits during ripening. Numbers under the *x*-axis indicate the days stored at RT after harvest; 0 signifies the commercial harvest date.

The fruits of ‘Nanguo’ pear are typical climacteric fruits. Their ripening process is different from those of *P. bretschneideri* and *P. pyrifolia*, most of which are nonclimacteric. Unlike European pear (*P. communis*), the fruit of ‘Nanguo’ pear does not require a low temperature before ripening. A burst of ethylene production occurred at approximately 7 days after harvest with storage at RT, and the fruit rapidly lost firmness during this process (Figure 1). Moreover, we measured the titratable acid and total soluble solids of the fruit juice of ‘Nanguo’ pear, which began to decrease at 15 d (Figure 1). Therefore, we sampled the fruits of ‘Nanguo’ pear at commercial harvest time, at the pre-climacteric stage (0, Pre) and fruits stored at RT for 10 d, which were at the post-climacteric stage (10, Post), using RNA sequencing to compare the transcriptomes of these two stages. Total RNA was extracted from these samples and reverse-transcribed into cDNA, which was then sequenced using the Illumina Genome Analyzer HiSeq 2000.

The RNA-seq generated 0.2 billion reads, each of which were 101 nucleotides long (paired-end), with approximately 6 billion nucleotides from each sample. The TopHat software [16] was used to map the reads to the reference pear genome. As shown in Table 1, uniquely mapped reads are defined as those reads that mapped to only one location in the genome, and multiple mapping reads are defined as those that mapped to more than one location in the reference genome; splice reads are those that span a splice junction. Approximately 61.06% of the reads were mapped to unique locations, and 10.38% of the reads were mapped to multiple locations. Perfectly matching reads accounted for 35.47% of the total reads.

**Table 1:** Number of reads sequenced and mapped using TopHat.

Sample	Sequenced reads	Total alignments	Perfectly matching reads	Unique mapping reads	Multiple mapping reads	Junction reads
Pre-1	60,030,036	36,944,508	12,840,015	32,948,223	3,996,285	6,784,280
Pre-2	63,724,323	38,247,274	13,424,314	34,277,275	4,278,342	6,678,242
Post-1	75,423,978	44,878,833	16,611,508	40,344,978	4,533,855	6,814,009
Post-2	74,259,754	43,472,116	16,272,415	38,572,438	4,235,241	7,824,353

### Identification of DEGs between Pre- and Post-Climacteric Reads

**Figure 2:**
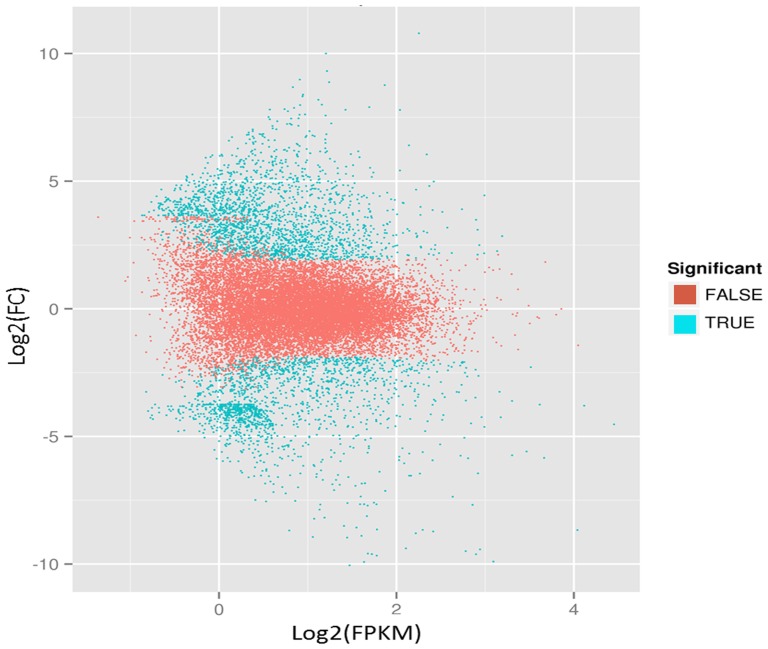
DEGs in the Pre and Post samples. Significantly up- or downregulated genes are marked in blue, and genes showing no significant differential expression are marked in red, using the threshold of FDR≤0.001 and log_2_Ratio≥1.

**Figure 3:**
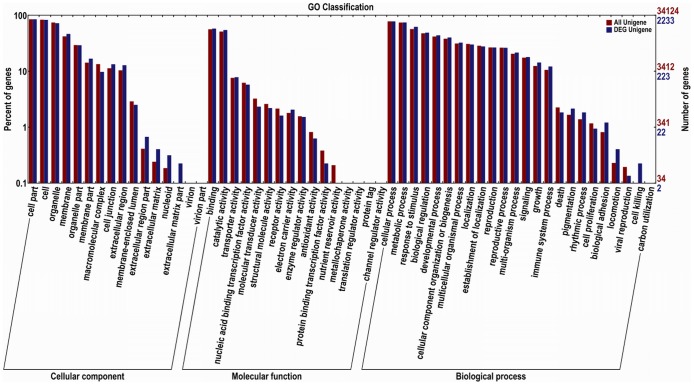
GO assignment of all DEGs. The unigenes were mapped to three main categories: cellular component, molecular function and biological process. The right-hand *y*-axis indicates the number of annotated unigenes.

To identify genes involved in fruit ripening, we used the EBseq software to compare the transcripts in Pre and Post samples [23]. The transcript abundance of each gene was estimated using FPKM. In total, 3,279 unigenes were differentially expressed (log2Ratio ≥1, false discovery rate (FDR) ≤0.005), among which the transcripts of 1,967 unigenes were more abundant in Pre samples, and 1,312 unigenes were more abundant in Post samples (Figure 2). To annotate these genes, BLASTx was applied to understand their biological function, with a cut-off *E* value of 10^−7^ (Table S1). GO analysis was employed to analyze the DEGs. Of the 3,279 DEGs, 2,587 were successfully categorized into GO groups (Figure 3). In the cellular component category, the greatest numbers of genes were found in the terms ‘cell part’, ‘cell’ and ‘organelle’. In the molecular function category, most of the DEGs were mapped into the ‘binding’ and ‘catalytic activity’ groups. In the biological process category, the largest groups were ‘cellular process’, ‘metabolic processes’ and ‘response to stimulus’.

### GO enrichment and Clusters of Orthologous Groups of protein (COG) analysis of DEGs

GO enrichment analysis was applied to analyze the DEGs. Within the ‘biological process’ group, 19 groups, including ‘oxidation-reduction process’, ‘defense response’, ‘polysaccharide biosynthetic process’ and ‘positive regulation of catalytic activity’, were significantly enriched (corrected *p* value ≤0.05) in the DEGs. Moreover, the ‘brassinosteroid biosynthetic process’ was also enriched, indicating its role in fruit ripening (Table 2; Table S2–S4). In the ‘molecular function’ group, only 5 groups were enriched; the most enriched group was glucan endo-1,3-beta-D-glucosidase activity. In the group ‘cellular component’, ‘plant-type cell wall’ group was the most enriched.

**Table 2:** Identification of over-represented GO terms in the DEG set.

GO terms	Number of elements in the whole transcriptome	Number of elements differentially expressed	Number of elements expected to be differentially expressed	Fold change	Corrected *p* value
**Biological Process**					
regulation of meristem growth	715	112	60.6	1.8	2.77E–07
microtubule nucleation	293	54	24.8	2.2	8.30E–05
cellular cation homeostasis	315	56	26.7	2.1	1.69E–04
oxidation-reduction process	2368	271	200.6	1.4	2.38E–04
sterol biosynthetic process	536	81	45.4	1.8	4.61E–04
defense response	1738	207	147.3	1.4	5.08E–04
polysaccharide biosynthetic process	461	72	39.1	1.8	5.95E–04
positive regulation of catalytic activity	248	46	21.0	2.2	6.61E–04
regulation of cell size	363	59	30.8	1.9	1.80E–03
brassinosteroid biosynthetic process	463	69	39.2	1.8	5.72E–03
plant-type cell wall organization	707	94	59.9	1.6	1.60E–02
plant-type cell wall cellulose metabolic process	205	37	17.4	2.1	1.69E–02
polysaccharide catabolic process	229	40	19.4	2.1	1.70E–02
response to far red light	426	63	36.1	1.7	1.93E–02
cell tip growth	508	72	43.0	1.7	2.11E–02
carbohydrate metabolic process	548	76	46.4	1.6	2.66E–02
cell wall pectin metabolic process	243	41	20.6	2.0	3.12E–02
response to cold	1810	203	153.4	1.3	3.57E–02
acetyl-CoA metabolic process	255	42	21.6	1.9	4.49E–02
**Molecular Function**					
glucan endo-1,3-beta-D-glucosidase activity	85	23	7.4	3.1	6.71E–04
symporter activity	27	12	2.3	5.1	1.01E–03
ADP binding	144	31	12.5	2.5	2.03E–03
2-alkenal reductase [NAD(P)] activity	457	65	39.6	1.6	5.94E–02
pyridoxal phosphate binding	168	29	14.6	2.0	3.03E–01
**Cellular Component**					
plant-type cell wall	1222	167	103.3	1.6	8.82E–08
anchored to plasma membrane	317	59	26.8	2.2	1.86E–06
plasmodesma	3857	408	325.9	1.3	1.25E–04
external side of plasma membrane	94	24	7.9	3.0	1.86E–04
integral to membrane	2672	282	225.8	1.2	1.12E–02
cell periphery	732	93	61.9	1.5	1.39E–02
extracellular region	2086	225	176.3	1.3	1.93E–02

**Figure 4:**
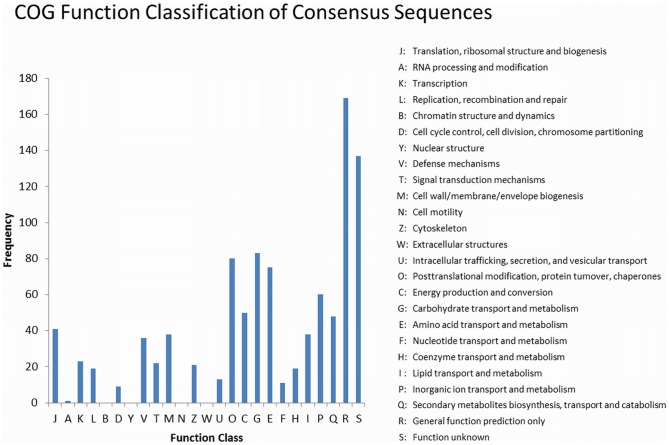
COG functional classification of DEGs.

In addition, the DEGs were subjected to COG analysis. Of the 3,279 DEGs, 993 were annotated in the COG database (Figure 4; Table S5). Most of the DEGs that were found were included in the COG category ‘Carbohydrate transport and metabolism’, followed by the category ‘Posttranslational modification, protein turnover, chaperones’ and ‘Signal transduction mechanisms’.

### Confirmation of RNA-seq results by qRT-PCR

**Figure 5:**
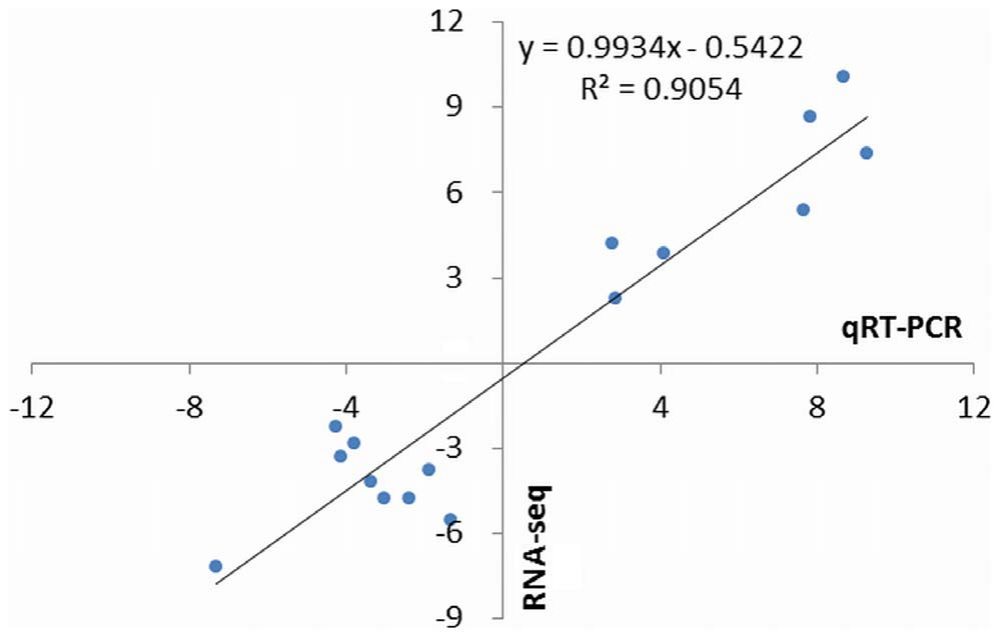
Correlation analysis of fold change data generated from qRT-PCR with that from RNA-seq. Sixteen genes were selected for qRT-PCR analysis.

To confirm the accuracy and reproducibility of the transcriptome analysis, 16 genes showing differential expression in the transcriptome analysis were selected for a qRT-PCR comparison of their expression levels between the Pre and Post samples. The scatterplot demonstrated a positive correlation between the log2 fold change determined by RNA-seq and qRT-PCR (Figure 5), thereby confirming our transcriptome analysis.

### Identification of DEGs related to plant hormones

**Figure 6:**
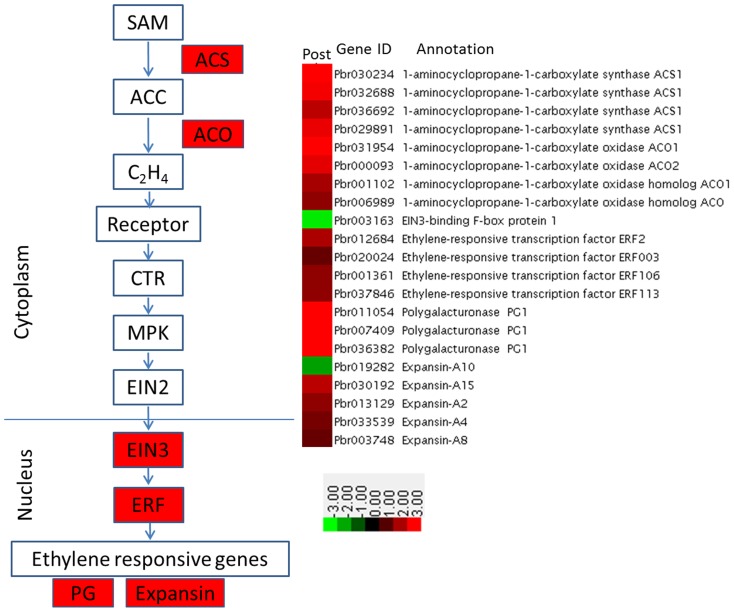
Heat map of relative expression levels for genes involved in ethylene biosynthesis and signaling.

Plant hormones are required for fruit development, maturation and ripening [24]. In our transcriptome analysis of Pre and Post samples, numerous identified DEGs were related to plant hormones, including ethylene, gibberellin, auxin and ABA as well as brassinosteroid (Table 3; Table S6). The role of ethylene in fruit ripening has been described in many studies. Among our data, many genes involved in the ethylene signaling pathway were identified as DEGs between the Pre and Post samples. Because several such genes were identified from other species, we constructed a heat map for the genes in the ethylene signaling pathway as markers to again confirm the accuracy of our RNA-seq data (Figure 6). Most of these genes, including *ACS*, *ACO*, *ERF*, *PG* and *Expansin*, showed higher expression in the Post samples, which was consistent with the previous reports [1], [10], [25].

**Table 3:** DEGs between the Pre and Post samples that are related to other plant hormone signaling pathways.

Gene ID	FDR	Log2 Post/Pre	Regulation	Annotation
**ABA**				
Pbr009089	0	5.4	Up	9-cis-epoxycarotenoid dioxygenase NCED1 [Phaseolus vulgaris]
Pbr039596	4.45E–04	2.3	Up	9-cis-epoxycarotenoid dioxygenase NCED1 [Phaseolus vulgaris]
Pbr006776	1.11E–04	2.2	Up	ABA 8′-hydroxylase 1 [Arabidopsis thaliana]
Pbr003860	5.22E–15	5.4	Up	ABA 8′-hydroxylase 2 [Arabidopsis thaliana]
Pbr025010	7.77E–07	−3.1	Down	Protein phosphatase 2C 16 (Precursor) [Arabidopsis thaliana]
**Auxin**				
Pbr021158	3.36E–03	2.2	Up	Indole-3-acetic acid-amido synthetase GH3 [Arabidopsis thaliana]
Pbr027098	7.12E–06	−3.4	Down	Auxin response factor 2 [Arabidopsis thaliana]
Pbr025597	7.34E–05	−2.6	Down	Auxin response factor 4 [Arabidopsis thaliana]
Pbr008550	3.44E–04	2.4	Up	Auxin response factor 17 [Arabidopsis thaliana]
**GA**				
Pbr009085	2.09E–12	4.2	Up	Gibberellin 2-beta-dioxygenase 1 [Pisum sativum]
Pbr036063	1.77E–10	3.8	Up	Gibberellin 3-beta-dioxygenase 2 [Arabidopsis thaliana]
Pbr011719	0	7.4	Up	Gibberellin 3-beta-dioxygenase 4 [Arabidopsis thaliana]
**BR**				
Pbr010897	3.94E–07	−3.1	Down	Cytochrome P450 90A1 [Arabidopsis thaliana]
Pbr016511	2.09E–03	−3.9	Down	Brassinosteroid-regulated protein BRU1 (Precursor) [Glycine max]

DEG analysis also identified genes involved in the signaling pathways of hormones other than ethylene. In the ABA signaling pathway, the finding that two genes for *NCED1*, which is the key enzyme in ABA biosynthesis, and two genes for *ABA 8*′*-hydroxylase*, which is essential for ABA dehydration, showed increases in expression in the Post samples suggests the importance of ABA in the ripening of climacteric fruits. Moreover, one gene encoding Protein Phosphatase 2C, which is also involved in the ABA signaling pathway, was found to be expressed at lower levels in the Post samples, and several genes encoding serine/threonine-protein kinases were also downregulated in the Post samples (Table 3; Table S6).

In the auxin signaling pathway, a *GH3* (*indole-3-acetic acid-amino synthase*) gene associated with the conjugation of IAA-Asp [3] was found to be upregulated in the Post samples, indicating its role in decreasing free IAA during fruit ripening (Table 3; Table S6). Moreover, three auxin response factor (ARF) genes showed up- or downregulation in the Post samples, indicating that they play different roles in fruit ripening (Table 3; Table S6).

Three genes encoding Gibberellin 2-beta-dioxygenase (GA2ox1) or Gibberellin 3-beta-dioxygenase (GA3ox4), which are responsible for the inactivation of GA, were observed to be highly expressed in the Post samples (Table 3; Table S6). These results indicated the involvement of GA in regulating fruit ripening.

In the BR pathway, one BR synthesis gene, *Cytochrome P450 90A1* (*CYP90A1*), was found to be downregulated in the Post samples, and brassinosteroid-regulated protein (BRU) was also lower in abundance in the Post samples, indicating its negative regulation of the fruit ripening process (Table 3; Table S6).

**Figure 7:**
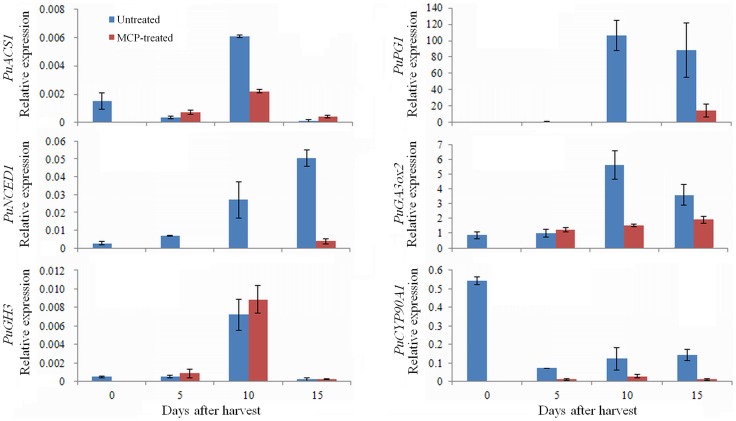
qRT-PCR analysis of genes involved in hormone-related pathways in fruit treated with or without 1-MCP during ripening. Numbers under the *x*-axis indicate the days stored at RT after harvest; 0 signifies the commercial harvest date.

To verify these results, six of the genes involved in these hormone signaling pathways were selected for qRT-PCR analysis, and their expression in 1-MCP-treated fruits was investigated (Figure 7). *PuACS1* (pbr032688), *PuPG1* (Pbr011054), *PuNCED1* (pbr009089), and *PuGA3ox2* (Pbr036063) were upregulated during fruit ripening, and their expression was inhibited by 1-MCP treatment. The expression of *PuGH3* (pbr021158) was increased at 10 d and decreased at 15 d, and its expression was not significantly affected by 1-MCP treatment. *PuCYP90A1* (Pbr010897) decreased in expression during fruit ripening and was slightly suppressed by 1-MCP treatment (Figure 7). The above results indicated that in addition to ethylene, other plant hormones are involved in regulating fruit ripening.

### Identification of DEGs related to transcription factors

Various transcription factors have been reported to be involved in fruit ripening [1], [26]. The best-known example is the *RIN* gene, which belongs to the *MADS-box* gene family. In our study, the *MADS-box* gene *AGL11* was downregulated in the Post samples, and its change in transcription might contribute to the ripening of pear fruit (Table 4).

**Table 4:** DEGs between the Pre and Post samples that are related to transcription factors.

Gene ID		FDR	Log2 Post/Pre	Regulation	Annotation
**MADS**					
Pbr011423		2.22E–04	−2.7	Down	AGAMOUS-like MADS-box protein AGL11 [Arabidopsis thaliana]
**NAC**					
Pbr026697		5.68E–04	−4.1	Down	NAC domain-containing protein 21 [Arabidopsis thaliana]
Pbr016205		4.44E–16	5.0	up	NAC domain-containing protein 8 [Arabidopsis thaliana]
Pbr020642		1.83E–04	2.4	up	NAC domain-containing protein 2 [Arabidopsis thaliana]
**WRKY**					
Pbr009057	3.29E–06		−3.0	down	Probable WRKY transcription factor 21 [Arabidopsis thaliana]
Pbr013092	1.15E–04		2.5	up	Probable WRKY transcription factor 33 [Arabidopsis thaliana]
Pbr032702	0		6.6	up	Probable WRKY transcription factor 56 [Arabidopsis thaliana]
Pbr000523	6.42E–03		2.0	up	Probable WRKY transcription factor 65 [Arabidopsis thaliana]
Pbr005390	1.35E–08		4.0	up	Probable WRKY transcription factor 69 [Arabidopsis thaliana]
Pbr042883	2.14E–07		3.4	up	Probable WRKY transcription factor 75 [Arabidopsis thaliana]
**HSF**					
Pbr002038	1.11E–06		3.9	up	Heat-stress transcription factor B3 [Arabidopsis thaliana]
Pbr036788	1.22E–15		−7.2	down	Heat-stress transcription factor A6b [Arabidopsis thaliana]

In addition to *MADS-box* proteins, other transcription factors, such as *NAC*, *WRKY* and *HSF*, were identified as DEGs between the Pre and Post samples (Table 4). Six *NAC* genes were downregulated and four were upregulated in the Post samples. *WRKY* transcription factors are primarily involved in the stress response, but five *WRKY* genes were upregulated and one was downregulated in the Post samples according to our data. Two *HSF* genes showed differential expression between the Pre and Post samples. Moreover, other transcription factors, such as *bHLH* and *MYB*, were observed to be differentially expressed (up or down) between the Pre and Post samples (Table S7), suggesting that they play regulatory roles in pear fruit ripening.

**Figure 8:**
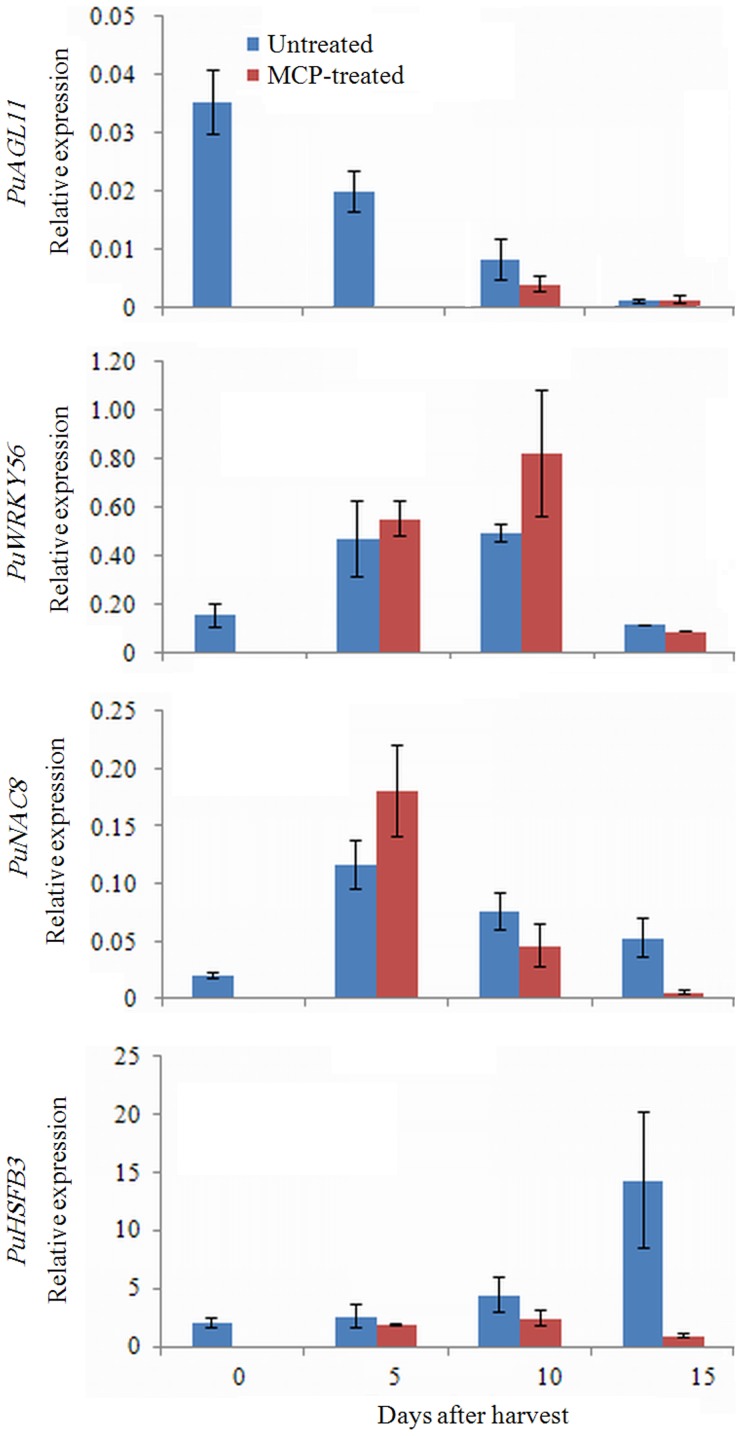
qRT-PCR analysis of transcription factor genes in fruit treated with or without 1-MCP during ripening. Numbers under the *x*-axis indicate the days stored at RT after harvest; 0 signifies the commercial harvest date.

One gene from each transcription factor family listed in Table 4 was selected for qRT-PCR validation. Their expression levels were consistent with the RNA-seq results. *PuAGL11* (pbr011423), *PuNAC8* (pbr016205) and *PuWRKY56* (Pbr032702) were not significantly influenced by 1-MCP treatment, whereas *PuHSFB3* (Pbr002038) was inhibited by 1-MCP (Figure 8).

### Identification of DEGs related to DNA methylation, cytoskeletal proteins and heat shock proteins (HSPs)

**Figure 9:**
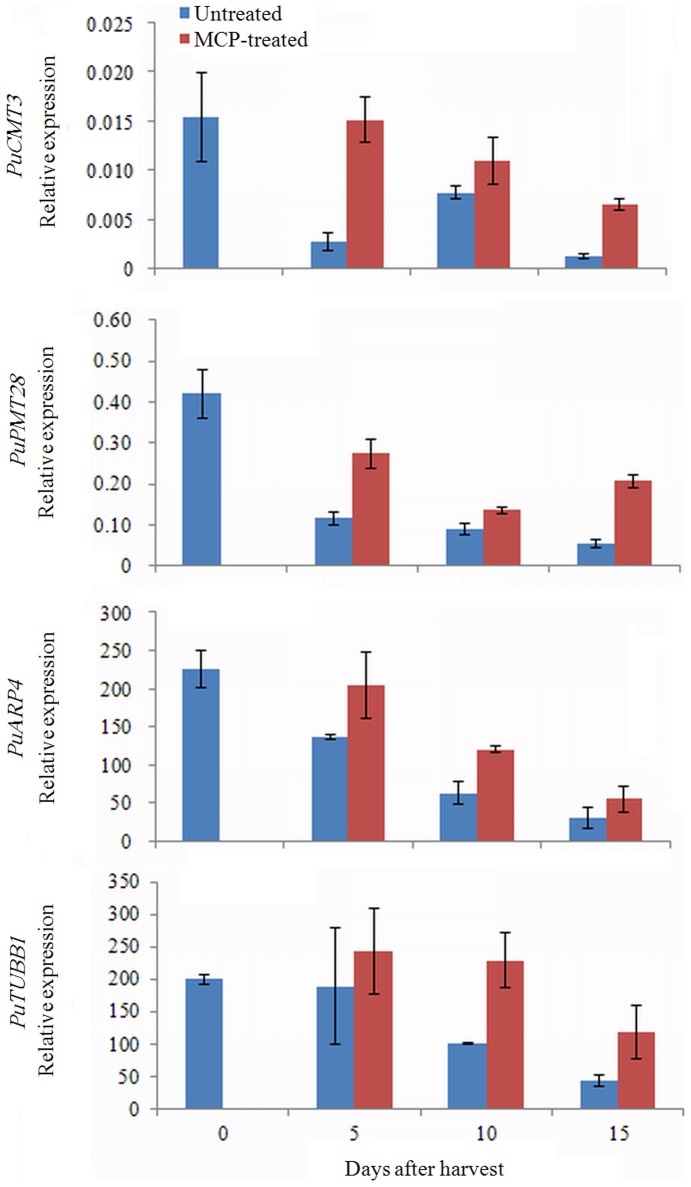
qRT-PCR analysis of genes related to DNA methylation and cytoskeletal proteins in fruit treated with or without 1-MCP during ripening. Numbers under the *x*-axis indicate the days stored at RT after harvest; 0 signifies the commercial harvest date.

In addition to the hormonal and transcriptional regulation, other factors were observed to be associated with fruit ripening. Genes encoding DNA (cytosine-5)-methyltransferase (CMT) and methyltransferase (MET) were downregulated in the Post samples (Table 5), suggesting that they contribute to the change of DNA methylation levels during fruit development and ripening. Moreover, two genes encoding cytoskeleton proteins, *ARP* (*actin regulated protein*) and *TUBB* (*tubulin beta-1 chain*), were downregulated in the Post samples (Table 5). The qRT-PCR results for *PuCMT3* (Pbr003336), *PuPMT28* (Pbr020117), *PuARP4* (Pbr009991) and *PuTUBB1* (Pbr035370) showed downregulated expression during pear fruit ripening, whereas 1-MCP treatment promoted their expression, implicating the involvement of DNA methylation and cytoskeletal proteins in fruit ripening (Figure 9). Furthermore, two HSP genes showed differential expression between Pre and Post (Table 5), demonstrating that HSP also participates in the process of fruit ripening.

**Table 5:** DEGs between the Pre and Post samples that are related to DNA methylation and cytoskeletal proteins.

Gene ID	FDR	Log2 Post/Pre	Regulation	Annotation
**DNA methylation**				
Pbr003336	1.51E–05	−3.0	Down	DNA (cytosine-5)-methyltransferase CMT3 [Arabidopsis thaliana]
Pbr020117	5.38E–08	−3.9	Down	Probable methyltransferase PMT28 [Arabidopsis thaliana]
**Cytoskeletal protein**				
Pbr009991	8.61E–04	−2.5	Down	Actin-related protein 4 ARP4 [Arabidopsis thaliana]
Pbr035370	1.82E–04	−2.5	Down	Tubulin beta-1 chain TUBB1 [Lupinus albus]
**Heat shock protein**				
Pbr007374	4.20E–04	2.4	up	Heat shock 70 kDa protein HSP70 [Glycine max]
Pbr007612	1.33E–08	−4.0	down	Heat shock 70 kDa protein HSP70 [Petunia hybrida]

## Discussion

Fruit ripening behavior is a very important trait that affects the fruit's shelf life, which is essential for determining the economic value of fruits after harvest. Much work has addressed the ripening behavior of climacteric fruit, in which ethylene has been known to play a critical role. In addition to an explosion of ethylene production following the respiratory climacteric, numerous other events are regulated, including the accumulation of sugars, acids, and pigments and the release of volatiles, for which thousands of genes change in expression. Although clear progress has been made in previous studies, a deeper understanding of the fruit ripening process is critical for improving the crop quality. In this study, we compared the fruit transcriptomes in pre- and post-climacteric fruits of a Chinese pear and found that 3’79 genes were differentially expressed after the respiratory climacteric. These genes were distributed among 31 GO terms, including ‘regulation of catalytic activity’, ‘oxidation-reduction process’ and ‘polysaccharide biosynthetic process’. Regarding the gene function, the DEGs are involved in the biosynthesis and signal transduction of ethylene and other plant hormones and include various transcription factors, DNA methylation and cytoskeletal proteins.

### Plant hormone signaling pathways and fruit ripening

Ethylene is well known to control the ripening of climacteric fruits [27]. Many studies have focused on genes involved in ethylene biosynthesis and signal transduction in the ripening of fruits such as tomato, apple and banana [27]–[31]. In our study, the transcriptome analysis revealed large numbers of DEGs in ethylene biosynthesis and signal transduction between the Pre and Post samples (Figure 6; Table S6).

Of note, our study also showed differential expression of genes related to other hormones. ABA has been proposed to play essential roles in the ripening processes of both climacteric and non-climacteric fruits [32]–[33]. Zhang et al. [34] reported that ABA is important for triggering ethylene biosynthesis in tomato fruit, during which time the expression of *LeNCED1*, a key gene in ABA biosynthesis, increases at the breaker stage. Moreover, the suppression of *LeNCED1* results in the downregulation of some ripening-related cell wall genes and a longer shelf life in tomato [5]. Consistent with this result, two pear *NCED1* genes were upregulated in the Post samples in our study (Table 3; Table S6). The shelf life of ‘Nanguo’ pear fruit was extended by 1-MCP treatment [14], and the expression of *PuNCED1* was also suppressed by 1-MCP in our data (Figure 7). These results suggest that ABA also acts as a critical factor and may interact with ethylene in the regulation of pear fruit ripening.

IAA (indole-3-acetic acid IAA) is the most common free form of auxin. It has been reported that the concentration of IAA declines prior to fruit ripening [2], [35]. However, IAA-aspartic acid (IAA-Asp), a conjugated form of IAA generated by GH3 (IAA-amido synthetase), increases in ripening fruits [3]. Moreover, the expression of *GH3* in grape berries increases at the onset of fruit ripening, and IAA treatment delays fruit ripening [3]. In our study, one *GH3* gene was upregulated in the Post samples (Table 3; Figure 7). These results suggest that *GH3* is important for maintaining a low level of IAA in mature fruit and promoting fruit ripening. However, the mechanism through which *GH3* interacts with the components of the ethylene pathway remains unclear because the application of 1-MCP slightly increased the expression of *GH3* (Figure 7). A recent study on an auxin response factor revealed that the downregulation of *SlARF4* in tomato gives rise to increased firmness and prolonged shelf life [36], suggesting that it promotes fruit ripening. In our study, *ARF17* was upregulated and the other two *ARFs* (*ARF2*, *ARF4*) were downregulated in the Post samples (Table 3; Table S6). These findings indicate that these *ARFs* act as positive or negative regulatory factors in pear fruit ripening; however, *ARFs* may instead be associated with sugar metabolism, as reported by Sagar et al. [37].

Little attention has been paid to the role of GA in fruit ripening. Three genes (*GA3ox2*, *GA3ox4*, *GA2ox1*) involved in the GA signaling pathway were upregulated in the Post samples in our study (Table 3; Table S6), indicating a role for GA in pear fruit ripening. Additionally, the expression of *PuGA3ox2* was inhibited by 1-MCP treatment (Figure 7), suggesting that GA participated in the ethylene signaling pathway during fruit ripening.

BR is involved in various physiological processes [38]. Here, one *BRU1* gene was observed to be downregulated in ripening pear fruits (Table 3; Table S6). A CYP90A1 gene involved in BR biosynthesis was downregulated in fruit ripening and was slightly suppressed by 1-MCP treatment (Figure 7). This result suggests that BR also participates in regulating fruit ripening, but it remains unclear whether BR interacts with ethylene. Exploring the mechanisms by which BR modulates fruit development and ripening and those by which it interacts with other hormones will be a promising area for future study.

### Transcriptional regulation of fruit ripening

The current understanding of the transcription factors involved in fruit ripening was gained from the characterization of a *MADS-box* (*RIN*) gene in tomato. The deletion of *MADS-RIN* greatly inhibits the ripening of tomato fruit [6]. More recent studies have revealed that MADS-RIN interacts with the promoters of more than 200 genes, including those involved in ethylene biosynthesis and signaling, cell wall metabolism, and carotenoid biosynthesis, as well as other transcription factors [7]. In our data set, one *MADS-box* gene, *PuAGL11*, was downregulated in the Post samples and was not significantly affected by 1-MCP (Table 4; Figure 8). Although *PuAGL11* is not a homolog of *RIN* (data not shown), its regulatory role in fruit ripening is worth identifying because of its differential expression.

The NAC domain family of transcription factors has been reported to participate in various biological processes, including fruit ripening [38]. Shan et al. [26] reported that two banana *NAC* transcription factor genes, *MaNAC1* and *MaNAC2*, interact with the *EIL* (*EIN3-like*) gene, which functions in the signaling pathway downstream of ethylene during fruit ripening. In tomato, the *NOR* (*non-ripening*) gene is also a member of the *NAC* transcription family, and it is considered to operate upstream of *MADS-RIN* [1]. In apple, the *NAC* transcription factor genes *MdNAC1* and *MdNAC2* interact with *MdRTE1* (*reversion-to-ethylene sensitivity 1*) [39]. Our data showed that two *NAC* genes were upregulated and one was downregulated in the Post samples (Table 4; Table S6). *PuNAC8* was upregulated and then downregulated during fruit ripening and was inhibited by 1-MCP (Figure 8). These results suggest that the upregulated *NACs* may regulate fruit ripening by interacting with the components of ethylene biosynthesis or the downstream components of ethylene signaling, whereas the downregulated *NACs* could interact with other components in fruit ripening or act as negative regulators of fruit ripening. Thus, it would be interesting to globally characterize the *NAC* domain transcription factors and their interactions with other components of fruit ripening.

A large number of studies have reported the involvement of WRKY transcription factors in the stress response. In our study, five *WRKY* genes were upregulated in the Post samples (Table 4), suggesting the association of the *WRKY* gene family with fruit ripening. *PuWRKY56* expression increased during fruit ripening and was promoted by 1-MCP, suggesting that it may regulate fruit ripening via factors other than ethylene (Figure 8). To our knowledge, there is no report discussing the function of *WRKY* genes in fruit ripening; therefore, research in this area would open a new line of inquiry and expand our knowledge of fruit ripening.

HSF is usually expressed in response to heat stress, but two *HSF* genes showed differential expression between the Pre and Post samples in our results (Table 4). Additionally, *PuHSFB3* demonstrated increased expression during ripening and was suppressed by 1-MCP treatment (Figure 8). This result indicates that *PuHSFB3* is involved in fruit ripening and may participate in the ethylene signaling pathway in this process.

The *MYB* and *bHLH* families of transcription factors were also observed to be differentially expressed (up- or downregulated) between the Pre and Post samples (Table S7). These functions have not previously been reported and, thus, require further study.

### DNA methylation, cytoskeletal proteins and HSPs in fruit ripening

DNA methylation regulating fruit ripening has been investigated through the characterization of *Cnr* locus in tomato, as discussed above. The hypermethylation of cytosine in the promoter region of the *SBP* gene inhibits the ripening of tomato fruit [11]. Zhong et al. [40] recently performed methylome analysis on tomato with a single-base resolution and revealed that the changes in methylation level during development play essential roles in initiating fruit ripening. This finding suggests the importance of investigating the roles of DNA methylation-related genes in fruit ripening. In our study, a *CMT3* gene encoding DNA (cytosine-5)-methyltransferase and a MET gene encoding methyltransferase showed lower expression in the Post samples (Table 5); both genes were downregulated during fruit ripening, and 1-MCP treatment increased their expression (Figure 9). These results indicate that these genes do not need to maintain a high methylation level with their higher expression because the demethylation during fruit ripening and the inhibition of the ripening process slowed the decrease in their expression. To our knowledge, this is the first report describing the involvement of the *CMT3* and *MET* genes in fruit ripening. Uncovering the mechanisms by which *CMT3* or *MET* regulate the DNA methylation level of related genes during the fruit ripening process would be quite useful, and more work must be performed to elucidate this topic.

Most of the cytoskeletal protein genes are housekeeping genes that show no transcriptional change during various biological processes. However, we showed that *PuARP4* and *PuTUBB1* were downregulated during fruit ripening and that 1-MCP treatment increased their expression (Table 5; Figure 9). This result suggests that ARP4 and TUBB1 are critical to maintaining the cell structure and fruit firmness during ripening, and their action may be regulated by ethylene. Because this is a new area in the study of fruit ripening, the component through which cytoskeleton proteins participate in the ethylene signaling pathway in regulating fruit ripening is of interest.

Medina-Escobar et al. [41] isolated cDNA fragments for HSPs from ripening strawberry fruit. Wang et al. [25] also reported that the *HSP* gene *MdHSP17.5* was increased in expression during apple fruit ripening. In our study, one *HSP70* gene was downregulated and another was upregulated in the Post samples (Table 5). These results indicate that these two *HSP* genes are associated with fruit ripening. HSPs are chaperone proteins and might enclose the ripening-related genes and release them for expression when a ripening signal is available. A very short period of heat shock can activate the expression of *HSPs*, which occurs mostly at noon, when the sunlight is highest in intensity. In addition, its expression may be brief and can be quite different between fruits exposed to sunlight and those under shadow. This complicates the study of the HSP regulation of fruit development and ripening, but the possible underlying mechanism is an extremely attractive hypothesis.

## Conclusions

In conclusion, our data suggest that in addition to ethylene, other hormones including ABA, auxin, GA and BR play important roles in regulating fruit ripening, during which they may engage in crosstalk with ethylene. The transcriptional regulation of fruit ripening involves several families of transcription factors, including *NAC*, *WRKY* and *HSF*, in addition to the *MADS* family. Furthermore, the DNA methylation-related genes *CMT3* and *MET* and the cytoskeletal genes *ARP4* and *TUBB1* are also involved in fruit ripening. Our results provide useful information and open new windows for research in pear fruit ripening.

## Supporting Information

Table S1 **Annotations of the DEGs.**
(XLSX)

Table S2 **List of GO enrichment analysis results: category 'biological process'.**
(XLSX)

Table S3 **List of GO enrichment analysis results: category 'molecular function'.**
(XLSX)

Table S4 **List of GO enrichment analysis results: category 'cellular component'.**
(XLSX)

Table S5 **COG analysis of DEGs.**
(XLSX)

Table S6 **List of DEGs between the Pre and Post samples that are related to plant hormone signaling pathways.**
(XLSX)

Table S7 **List of DEGs between the Pre and Post samples that are related to other transcription factors.**
(XLSX)

Table S8 **List of primers used for qRT-PCR analysis in this study.**
(XLSX)
